# Bronchoalveolar lavage (BAL) cells in idiopathic pulmonary fibrosis express a complex pro-inflammatory, pro-repair, angiogenic activation pattern, likely associated with macrophage iron accumulation

**DOI:** 10.1371/journal.pone.0194803

**Published:** 2018-04-12

**Authors:** Jungnam Lee, Ivan Arisi, Ermanno Puxeddu, Lazarus K. Mramba, Massimo Amicosante, Carmen M. Swaisgood, Marco Pallante, Mark L. Brantly, C. Magnus Sköld, Cesare Saltini

**Affiliations:** 1 Division of Pulmonary, Critical Care and Sleep Medicine, University of Florida College of Medicine, Gainesville, Florida, United States of America; 2 Genomics Facility, European Brain Research Institute, Rome, Italy; 3 Department of Biomedicine and Prevention, University of Roma “Tor Vergata”, Rome, Italy; 4 Department of Medicine, University of Florida College of Medicine, Gainesville, Florida, United States of America; 5 Department of Medicine, Respiratory Medicine Unit, and Lung-Allergy Clinic, Karolinska University Hospital Solna, Stockholm, Sweden; Universitatsklinikum Freiburg, GERMANY

## Abstract

Idiopathic pulmonary fibrosis (IPF) is a chronic lung disease of unknown cause characterized by alveolar epithelial damage, patchy interstitial fibrosis and diffuse microvascular abnormalities. In IPF, alveolar clustering of iron-laden alveolar macrophages—a common sign of microhemorrhage, has been associated with vascular abnormalities and worsening of pulmonary hypertension. As iron-dependent ROS generation has been shown to induce unrestrained macrophage activation in disease models of vascular damage, we explored alveolar macrophage activation phenotype in IPF patients (n = 16) and healthy controls (CTR, n = 7) by RNA sequencing of bronchoalveolar lavage (BAL) cells. The frequencies of macrophages in BAL cells were 86+4% and 83.4+8% in IPF and CTR groups, respectively (p-value = 0.41). In IPF patients, BAL cells showed increased iron-dependent ROS generation (p-value<0.05 vs CTR). Gene expression analysis showed overrepresentation of Gene Ontology processes/functions and KEGG pathways enriched in upregulated M1-type inflammatory (p-value<0.01), M2-type anti-inflammatory/tissue remodeling (p-value<0.0001), and MTPP-type chronic inflammatory/angiogenic (p-value<0.0001) chemokine and cytokine genes. The *ex vivo* finding was confirmed by the induction of iron-dependent ROS generation and chemokine/cytokine overexpression of Ccl4, Cxcl10 (M1), Il1rn (M2), Cxcl2, and Cxcl7 (MTPP) in MH-S murine immortalized alveolar macrophages exposed to ferric ammonium citrate in culture (p-value<0.05 vs CTR). The data show alveolar macrophage expression of a pro-inflammatory, tissue remodeling and angiogenic complex activation pattern, suggesting that iron accumulation may play a role in macrophage activation.

## Introduction

Idiopathic pulmonary fibrosis (IPF), a progressive interstitial pneumonia of unknown cause disproportionately affecting older adults, is defined by the histopathological pattern of usual interstitial pneumonia (UIP) that is characterized by patchy distribution of fibrosis areas with peripheral honeycombing and scattered fibroblastic foci resulting from microscopic centers of acute alveolar epithelium injury of yet unrecognized cause [1].

The role of alveolar macrophage-driven lung inflammation in the pathogenesis of IPF has been long debated [2]. A number of bronchoalveolar lavage (BAL) studies have evaluated human alveolar macrophage activation in IPF using immunochemistry, immunocytochemistry, proteomics, RT-PCR or RNA microarrays to assess the production of cytokines and chemokines with pro-inflammatory or pro-repair/fibrogenic activities. These studies reported the expression of a number of chemokines appertaining to the “classic” M1 pro-inflammatory activation phenotype, as MIP-1α (CCL3), MIP-1β (CCL4), MIP-3α/LARC (CCL20) or to the “alternative” M2 anti-inflammatory, pro-fibrotic activation phenotype as the MCP-1 (CCL2), MCP-4 (CCL13), MIP-4/PARC (CCL18), Eotaxin-2 (CCL24) [3–5]. In addition, other studies reported the production of pro-inflammatory/angiogenic chemokines, including GROα (CXCL1), ENA78 (CXCL5), PPBP (CXCL7) and IL-8 (CXCL8) [4, 6]. In particular, Schupp and colleagues reported the production of anti-inflammatory/pro-fibrogenic chemokines CCL2, CCL17, CCL18 and CCL22, concomitantly with the pro-inflammatory chemokines CXCL1 and IL-8 by BAL alveolar macrophage in IPF exacerbation [3].

Recent studies in murine models of pulmonary fibrosis have shown that alveolar macrophages play a key role in fibrosis pathogenesis [7]. In particular, Misharin et al. showed that both monocyte-derived alveolar macrophages and tissue-resident alveolar macrophages up-regulated both M1 and M2 genes in response to bleomycin, with higher expression of a number of both M1 and M2 genes in monocyte-derived macrophages although without a clear shift toward either phenotype [8].

Histopathological and BAL IPF studies have shown that increased numbers of iron-laden macrophages cluster in the alveolar and interstitial spaces in a significant proportion of affected individuals. In particular, alveolar macrophage iron accumulation has been described in IPF, in association with increased capillary density [9], pulmonary veno-occlusive disease [10], microvasculitis, [10, 11] and pulmonary arterial hypertension [10, 12, 13]. Iron accumulation can drive macrophage generation of reactive oxygen species (ROS) and a pro-inflammatory activation that persists, in the presence of concomitant expression of anti-inflammatory/pro-repair cytokines, leading to “unrestrained”, non-resolving inflammation, as shown in varicose vein skin ulcer [14], haemophilia hemarthrosis [15] and spinal cord injury [16] studies.

In this context and with the background of the above IPF macrophage studies [9–13, 17], the present study was designed to explore the activation phenotype of BAL macrophages in IPF and its possible relation to iron accumulation.

## Materials and methods

Sixteen patients (all Caucasians, 4 females) diagnosed according to international guidelines by multidisciplinary discussion (MDD). HRCT scans were evaluated systematically according to the ATS/ERS/JRS/ALAT guidelines [1], including multiplanar HRCT chest scans with standard and Minimum Intensity Projection algorithms for improved detection of ground-glass opacities, linear attenuation and honeycombing patterns of traction bronchiectasis [18]. At the time of evaluation, 14 patients had HRCT definite UIP pattern. Two patients with possible UIP underwent lung biopsy with histologically probable UIP. The final MDD diagnosis was IPF. The GAP index was calculated according to Ley et al. [19]: 4 patients were stage I, 10 stage II and stage III. Eleven of the IPF affected individuals reported comorbidities (average = 2, Min 0, Max 5), including gastro esophageal reflux (31.3%), arterial hypertension (56.3%), pulmonary arterial hypertension (18.8%), coronary artery disease (6.2%), arrhythmia (6.3%), dyslipidemia (25%), diabetes type II (12.5%), hepatitis B (6.3%), depression (6.3%), and enlarged prostate (12.5%). At the time of evaluation 3 patients were not receiving any medications, 2 were taking acetylsalicylic acid alone, and one N-acetyl cysteine alone. The other 10 patients received different combination of the above drugs with proton pump inhibitors, statins and anti-hypertensive drugs for a total of 7 patients under proton pomp inhibitors therapy, 6 under acetylsalicylic acid, 6 statins and anti-hypertensive drugs. No patient was under treatment with immune-suppressants or antianticoagulants. Subjects were evaluated, after written informed consent was obtained (Policlinico Tor Vergata Ethics Committee, Rome, Italy, n.138/13).

Seven healthy controls (all Caucasian, 4 females) underwent fiberoptic bronchoscopy (FOB) and BAL under an investigational protocol (Policlinico Tor Vergata Ethics Committee, Rome, Italy: SCS/REDD/08 n.123/08) after written informed consent.

BAL of controls and IPF patients was carried out using standard procedures, according to ATS guidelines [20], as previously described [17]. Cell differentials were counted on May-Grunwald stained cytopreparations and iron macrophage accumulation was assessed by Prussian blue stain and scored according to Golde et al [17, 21]. All IPF patients included in the study had BAL macrophage differential counts >80%, cell viability, by trypan blue vital stain, was >95%; epithelial cells contamination was <5% and red blood cell contamination was negligible (Table 1).

**Table 1:** Study population characteristics of IPF patients and controls undergoing fiberoptic bronchoscopy with BAL.

	Control*	IPF patients*	Pvalue†
N	7	16	
Age	46.9 (±6.23)	67 (±8.62)	0.001
Gender (Male ⁄ Female)	3 ⁄ 4	12 ⁄ 4	0.182
Current/Ex/Never smokers	6 ⁄ 0 ⁄ 1	4 ⁄ 10 ⁄ 2	1.000^$^
Smoke history (pack years)	19.3(±13.06)	21.2(±16.34)	0.806
FVC (%)	115.05(±16.72)	80.6(±27)	0.009
Dlco (%)	82.06(±7.59)	48.6(±16)	0.001
BAL cell count			
Macrophages (%)	83.4 (±7.68)	86.1 (±3.84)	0.671
Neutrophils (%)	3.5 (±0.90)	7.3 (±4.04)	0.019
Lymphocytes (%)	8.3 (±8.31)	3.0 (±2.49)	0.047
Eosinophils (%)	1.2 (±1.70)	3.0 (±1.87)	0.026
Basophils (%)	0.0 (±0.00)	0.0 (±0.12)	0.557

Definition of abbreviations: FVC = Forced Vital Capacity, Dlco = Diffusing Capacity,

*Data is expressed as mean and standard deviation

^†^Comparisons between the groups were performed by Mann-Whitney test or Fisher’s exact test for frequencies

^$^Current and ex-smokers compared to never smokers

Generation of iron-dependent ROS was measured by CM-H2DCFDA (Invitrogen, Molecular Probes, Carlsbad, CA, USA) fluorescence on fresh, unstimulated BAL cells as spontaneously generated fluorescence that could be inhibited by the iron chelator deferiprone (DFP, Sigma Aldrich, St Louis, MO, USA). BAL cells (1x10^5^) were washed twice in PBS, and incubated in RPMI without phenol red (Sigma Aldrich) in the dark for 1 hr (37°C, 5% CO2) with/out 100 μM DFP. CM-H2DCFDA (5 mM) was then added and fluorescence measured after 1 h incubation (LS50B LuminSpectrometer, Perkin Elmer, Waltham, MA, USA) as described [17].

### RNA sequencing and analysis

Total RNA was obtained from un-fractionated BAL cells (5x10^6^ cells), kept on ice until RNA extraction (RNA/DNA/Protein Purification Kit, Norgen Biotech Corp., Thorold, ON, Canada). As previous studies showed generalized gene under-expression in IPF macrophages [22], libraries were prepared with the FFPE RNA-seq system (NuGEN Technologies, San Carlos, CA) [23], as recommended for clinical samples potentially affected by low RNA integrity [23]. The 260/280 nm ratio was 2.05±0.05 using NanoDrop (Thermo Scientific, Wilmington, DE, USA). Total RNA was quantified using a Qubit RNA High Sensitivity Assay (invitrogen, ThermoFisher Scientific, Waltham, MA, USA) and RNA integrity number (R.I.N.) was measured using an Agilent 2100 Bioanalyzer with the Total RNA pico kit (Agilent Technologies, Santa Clara, CA, USA). RINs of controls and IPF patients were on average 6.3±2.55 and 5.4±2.02, respectively. Libraries were prepared, as above, and fragmented to an average size of 200 bp using the Covaris S2 (Covaris, Woburn, MA, USA). Fragmented cDNA was end-repaired, A-tailed and ligated with barcoded Illumina adapters. Ligated DNA was treated with NuGEN mouse Insert Dependent Adaptor Cleavage (InDA-C) reagent to deplete cDNA corresponding to ribosomal RNA, and PCR amplified. Final library yield was measured using the Qubit High Sensitivity DNA assay (Thermo Fisher Scientific) and library size assessed using the Agilent 2100 Bioanalyzer (Agilent Technologies, Santa Clara, CA, USA). Libraries were then pooled, and sequenced using single-end 75 bp reads on the Illumina NextSeq500 (Illumina, San Diego CA, USA). In average, 82,247,297 ± 27,317,568 reads were generated of which 69,860,529 ± 24,683,386 (84.51 ± 1.58%) were uniquely aligned (Cofactor Genomics, Saint Louis, MO, USA). Raw sequence data in FASTQ format were assessed for Per Base Sequence Quality (Phred average score = 33, ribosomal RNA content < 2%) (Cofactor Genomics, Saint Louis, MO, USA).

Raw read counts were mapped on UCSC mRNA transcript human database based on the GRCh37/hg19 version of the Genome Reference Consortium consensus representation of the human genome using the NovoAlign tool. Raw counts per transcript were then aggregated onto unique UCSC gene symbols (26,605 unique gene symbols). Raw counts were filtered by keeping only protein-coding genes satisfying the following condition: Counts Per Million (CPM) > 1.0 in at least 4 samples. The final data table comprised 19,696 gene symbols. Data are publicly available from the Gene Expression Omnibus database, https://www.ncbi.nlm.nih.gov/geo/query/acc.cgi?acc=GSE79544.

Normalization of RNA-sequences was obtained using the upper-quartile method provided by EDASeq package in R Bioconductor, followed by removing unwanted variation (RUV) technique using RUVg function provided by R Bioconductor package RUVSeq, where a set of 20 house keeping genes were used as negative controls [24]. RUVg has the potential to normalize RNA-sequences using housekeeping genes to successfully remove unwanted effects thereby producing an accurate evaluation of gene differential expression. After removing the unwanted variation, the process of looking for differentially expressed genes was accomplished using the generalized linear model with a negative binomial distribution (GLM) in using the edgeR Bioconductor package [25]. This was done by considering a design matrix that includes diagnosis status, the factors of unwanted variation and the normalized counts obtained by regressing the original counts on the unwanted factors [25]. Differential gene expression analysis between IPF and CTR was done using the exactTest function in EdgeR Bioconductor package, to generate a differential expression gene table using a cutoff of False Discovery Rate (FDR) < 0.05, for statistical significance and log2FC > 1.0, resulting in 88 differentially expressed genes (Table A in S1 File).

Gene Ontology and Pathway analysis of differentially expressed genes in IPF, was performed using the iPathways web resource (http://www.advaitabio.com/ipathwayguide) with the Gene Ontology (GO) Consortium database (Release 09/19/2014) and the (Kyoto Encyclopedia of Genes and Genomes, KEGG, database, Release 78.0+/06-02, Jun 16) [26], using the 88 gene symbol differential expression gene table (see above).

For Biological Processes and Molecular Functions analysis, GO term enrichment was ranked according to P-values computed by the hypergeometric distribution and the elim pruning method [27]. For Pathway analysis KEGG pathway enrichment was assessed using the Impact Analysis method and scored by Fisher’s exact test Pvalues, corrected for multiple comparisons (FDR) [28].

To assess the expression of macrophage activation-specific gene markers, we compiled gene lists from published studies of *in vitro* and *in vivo* activated polarized human monocyte-derived macrophages (MDM) and macrophages. The enrichment of M1, M2 and MTPP markers was identified by comparing the differential expression gene table (FDR<0.05, log2FC>1.0, Table A in S1 File) with the M1-type gene list and the M2-type gene lists obtained by Becker M. et al. (Becker M. et al. Sci Rep 2015: S2 and S3 Tables, respectively [29]) using monocyte derive macrophages (MDM) stimulated with interferon-γ and LPS or interleukin-4, respectively. In addition, the above differential expression gene table (Table A in S1 File) was compared to the MTPP-type gene list obtained by Xue J. et al. (Immunity 2014: Table S2B [30]) using macrophage-colony stimulating factor-differentiated MDMs, stimulated with TNF/Pam3CSK4/prostaglandin E2. Significant enrichment was assessed by Fisher exact test on a contingency table, using all the 19,696 protein coding genes identified in our study as the reference genome.

### Gene expression validation by qRT-PCR

Total RNA (1 μg), extracted from human BAL cells (86+4% macrophages in IPF and 83+4% in CTR) or murine immortalized alveolar macrophages, was reverse-transcribed using random hexamers and SuperScript^®^ VILO Master Mix (Invitrogen, 11755050), according to the manufacturer’s instruction. Quantification of PCR products was performed with 7500 Fast Real-time PCR (Applied Biosystems, Foster City, CA, USA). SYBR^®^ Select Master Mix (Applied Biosystems, Foster City, CA, USA) was used to produce fluorescence-labeled PCR products and to monitor increasing fluorescence during repetitive cycling of the amplification reaction. Primers specific for the CCL4, CXCL10, CCL13, CCL24, IL1RN, CXCL7/PPBP (CXCL7), CXCL8/IL-8 (CXCL8) transcripts, and for the GNB2L1 gene, as internal control, were designed using Primer-BLAST (http://www.ncbi.nlm.nih.gov/tools/primer-blast) and Primer3 (http://bioinfo.ut.ee/primer3-0.4.0/primer3). Gene expression levels of human chemokines and cytokines, indicated as corrected ΔCt values, were obtained by subtracting the GNB2L1 (housekeeping gene) raw Ct from the test genes raw Ct to obtain the classical ΔCt, then the absolute minimum value for GNB2L1 (the same for all samples, thus a constant value) was further added to the same, to rescale data. As for gene expression levels of mouse M1/M2 cytokines, the relative fold-induction vs. controls was calculated using the classical 2^ΔΔCt method [31]. Primer sequences are shown in Table B in S1 File.

### Gene expression validation by BAL fluid protein immunochemistry

To validate BAL macrophage expression of up-regulated chemokine genes, CXCL5, CXCL10, CCL13, CCL18, and PPBP/CXCL7 proteins were quantified on un-concentrated BAL fluid. CXCL-5 was measured using a Chemokine Human 5-Plex Panel II for the Luminex 100/200 platform, according to manufacturer instructions (Invitrogen, Thermo Scientific, Frederick, MD, USA). Enzyme-linked immunosorbent assay (ELISA) kits were used to measure CCL13, CCL18, CXCL-7 (Thermo Scientific, Frederick, MD, USA) and CXCL-10 (Invitrogen), according to the manufacturer’s protocol.

### *In vitro* modeling of alveolar macrophage iron overload

Immortalized murine alveolar macrophages, derived from a BALB/C strain mouse, (MH-S, CRL-2019, American Type Culture Collection, Manassas, VA) were cultured in a six-well plate to confluency in complete medium (RPMI 1640, 10% heat-inactivated fetal bovine serum, penicillin-streptomycin, 50 μM 2-meraptoethanol, 37 °C, 5% CO2) and treated with ferric ammonium citrate [C6H8FeNO7 dissolved in water] (FAC, 10μM, 100μM and 250μM) for 24, 72, 120 hours. Macrophage iron accumulation was assessed using the Prussian Blue Stain and staining intensity quantified by the Golde score as previously described [17]. ROS generation was determined by CM-H2DCFDA fluorimetry (3 X 10^4^ cells/well on a 96-well microplate, 37 °C, 30 min, 10 μM CM-H2DCFDA, Sigma C6827) as previously described [17] using a fluorescence plate reader (SpectraMax M3 ROM v3.0.22) at EX/Em = 485/535 nm in end point mode. For iron chelator assay, MH-S cells were treated with 30 μM of FAC for one day. The cells were washed with PBS and incubated with 100 mM of DFO at 37°C for one hour. The DFO-treated cells were incubated with 5 mM of CM-H2DCFDA at 37°C for 30 min, prior to reading with fluorescence plate reader.

Macrophage activation phenotype was assessed by quantitative PCR (qPCR) analysis of the expression of M1, M2, and MTPP genes Ccl4, Cxcl2, Cxcl10, Il1rn, and Cxcl7.

### Statistical analysis

Study population and *in vitro* study data are expressed as mean and standard deviation or percentage as appropriate. Comparisons between groups were made by using the Fisher’s exact test (for contingency tables), or non-parametric test, Mann-Whitney test and one way ANOVA with Scheffe multiple comparison. A p-value <0.05 was considered significant. All analyses were performed using the GraphPad Prism 6.0 (GraphPad software, San Diego, CA, USA) software package.

Normalization of RNA sequence data was carried out using the upper-quartile method, followed by RUV technique, using housekeeping genes (HKG) to remove unwanted effects and generate accurate estimation of gene fold change using GLM with a negative binomial distribution as described above.

## Results

### Alveolar macrophage activation phenotype analysis

**Fig 1:**
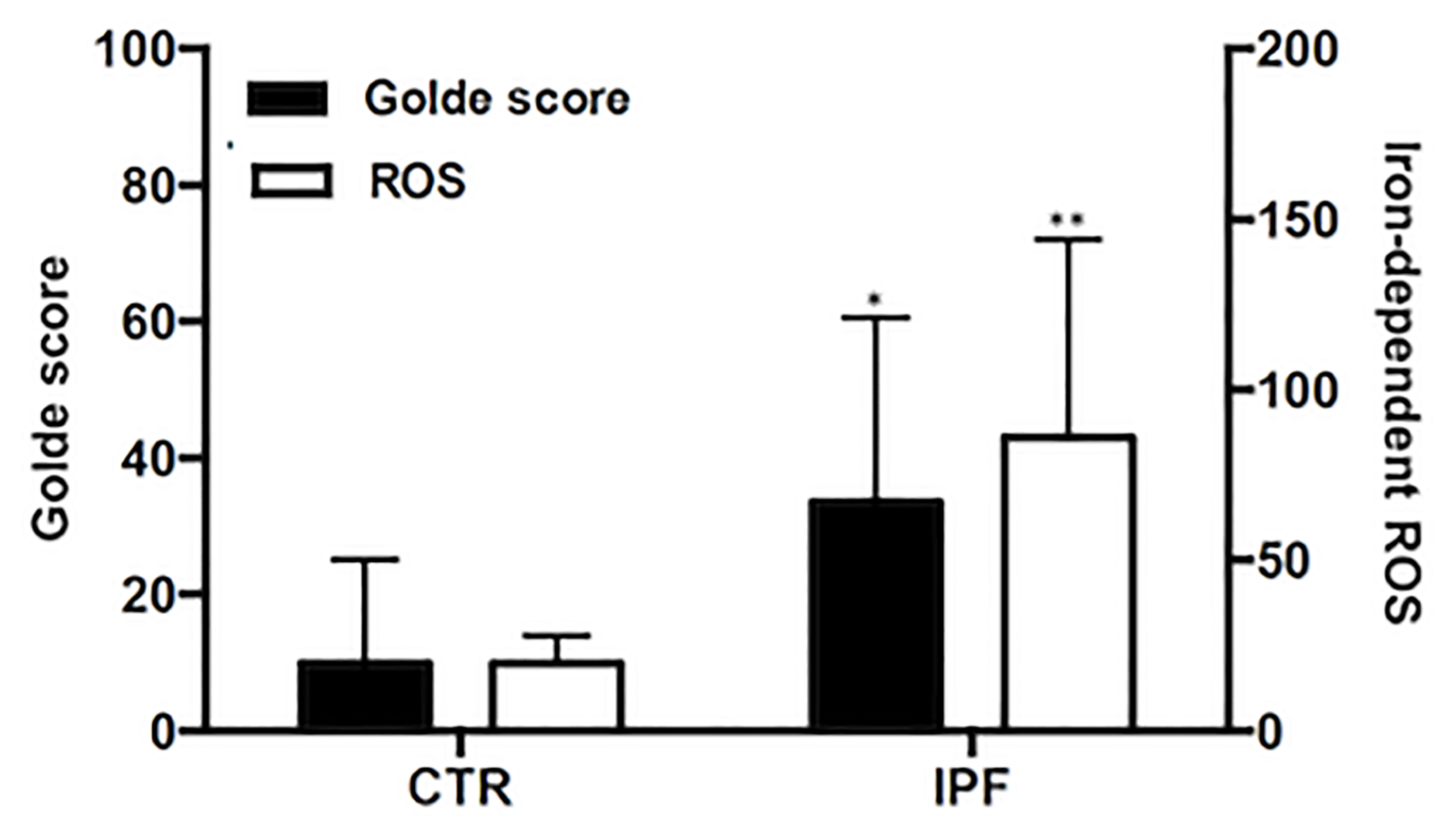
Intracellular iron and ROS levels in BAL cells. Alveolar macrophage intracellular iron was assessed using the Prussian Blue Iron Staining and the Golde score was calculated based on the staining intensity 14 IPF patients and 7 controls. Reactive oxygen species (ROS) were measured in 11 IPF patients and 7 controls, using CM-H2DCFDA fluorimetry. Shown are Golde scores and chelatable iron-dependent ROS levels per 10^5^ BAL cells in healthy controls and IPF affected individuals.

Alveolar macrophages recovered by BAL from IPF patients (86.1 ± 3.84% of total BAL cells, controls 83.4 ±7.68) were characterized by significant iron accumulation, as measured by Golde score (33.5 ± 27%, controls 9.9 ± 15.2, p<0.05, Fig 1). Analysis of the larger set of healthy control and IPF BAL cytopreparations described in the Supplementary information, using the Prussian blue stain and the Golde score showed that iron accumulation was significantly higher in IPF compared to healthy individuals (p<0.001, part A in S1 Fig), and was not significantly associated with subject’ age (control: r = 0.24, p = 0.26: IPF: r = -0.04, p = 0.74, part B in S1 Fig). In addition, macrophage Prussian blue staining was not associated with exposure (pack years) to tobacco smoke (control: r = 0,04, p = 0,83; IPF: r = 0,09, p = 0,47, data not shown). Finally, IPF BAL cells were characterized by significantly increased iron-dependent ROS generation (p<0.01, Fig 1).

**Fig 2:**
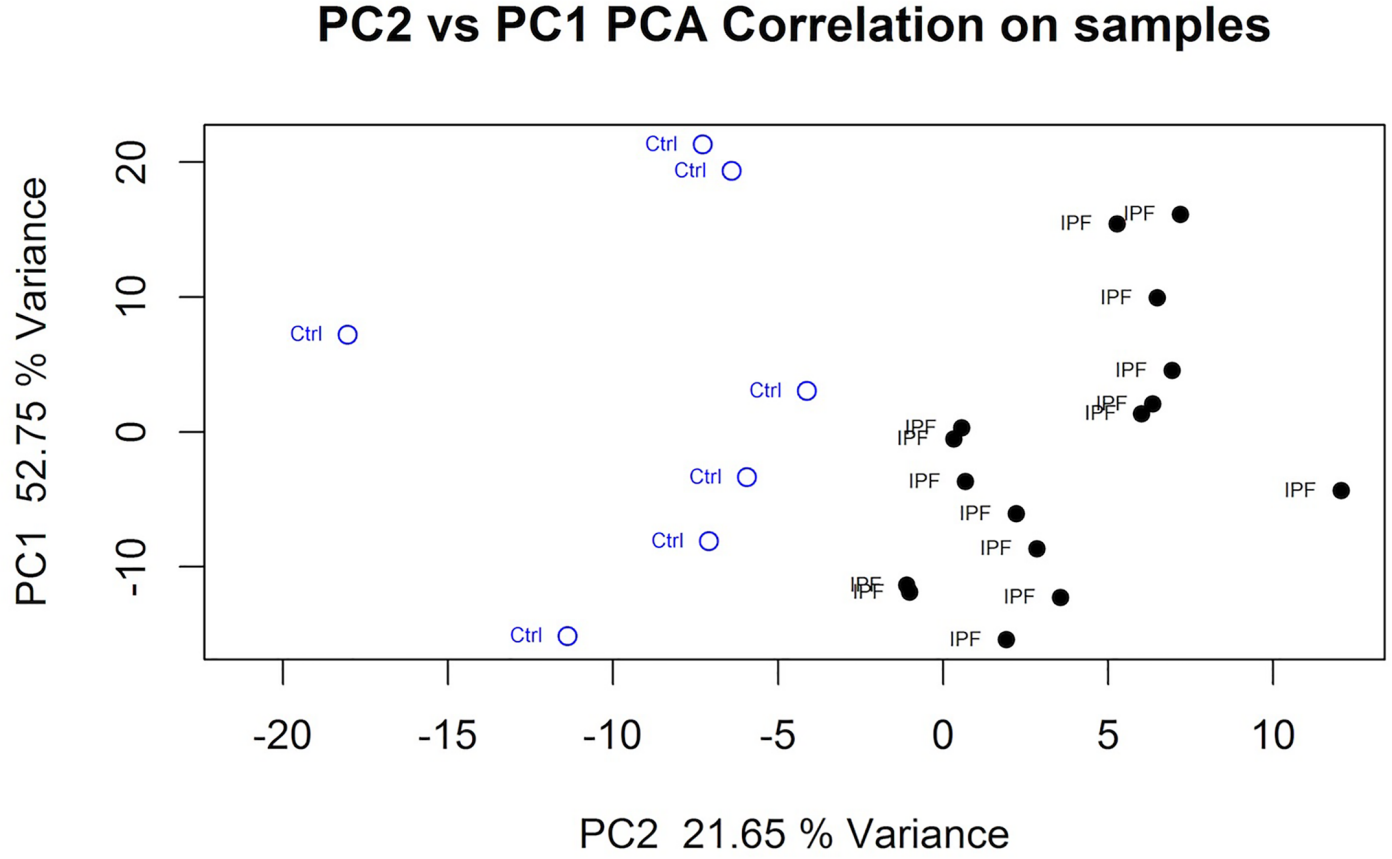
Principal Component Analysis of healthy controls and IPF patients. The PCA plot was computed using the differentially expressed gene list (FDR<0.05, log2FC>0.6 filter).

Using the differential expression gene table (see above), Principal Component Analysis showed separation between healthy control and IPF patient BAL macrophage gene expression (Fig 2). Based on the RNA sequence differentially expressed gene symbol list (FDR<0.05, log2FC>1, n = 88) Gene Ontology identified, “Chemokine-mediated signaling pathway” and “Chemokine activity” (Table 2) as the most significantly enriched Biological Process and Molecular Function.

**Table 2:** Gene Ontology (GO) term and KEGG pathway analysis.

**Category**	**Term**	**Description**	**CountD**	**CountAll**	**pv_elim**
GO Biological Process	GO:0070098	Chemokine-mediated signaling pathway	12	76	6.40E-16
	GO:0030593	Neutrophil chemotaxis	10	80	5.10E-09
	GO:0071347	Cellular response to interleukin-1	8	83	5.60E-08
GO Molecular Function	GO:0008009	Chemokine activity	12	46	1.60E-18
	GO:0045236	CXCR chemokine receptor binding	6	17	8.40E-08
	GO:0031720	Haptoglobin binding	3	3	1.00E-07
KEGG Pathway*				**OGC**	**pv_fdr**
	KEGG:4060	Cytokine-cytokine receptor interaction		14	5.60E-06
	KEGG:4062	Chemokine signaling pathway		10	5.60E-05
	KEGG:4668	TNF signaling pathway		7	6.45E-03

Definition of abbreviations: CountDE = the number of differently expressed genes belonging to the respective pathway, CountAll = the total number of genes belonging to the respective pathway, pv_*elim* = GO term’s P-value computed by the hypergeometric distribution and the *elim* pruning method (see Methods). OGC = Observed Gene Count. pv_fdr = False discovery rate (FDR): Fisher’s exact test P-value, corrected for multiple comparisons.

*KEGG pathways were assessed using both the iPathway (see Methods) and the String (http://string-db.org) tools.

**Fig 3:**
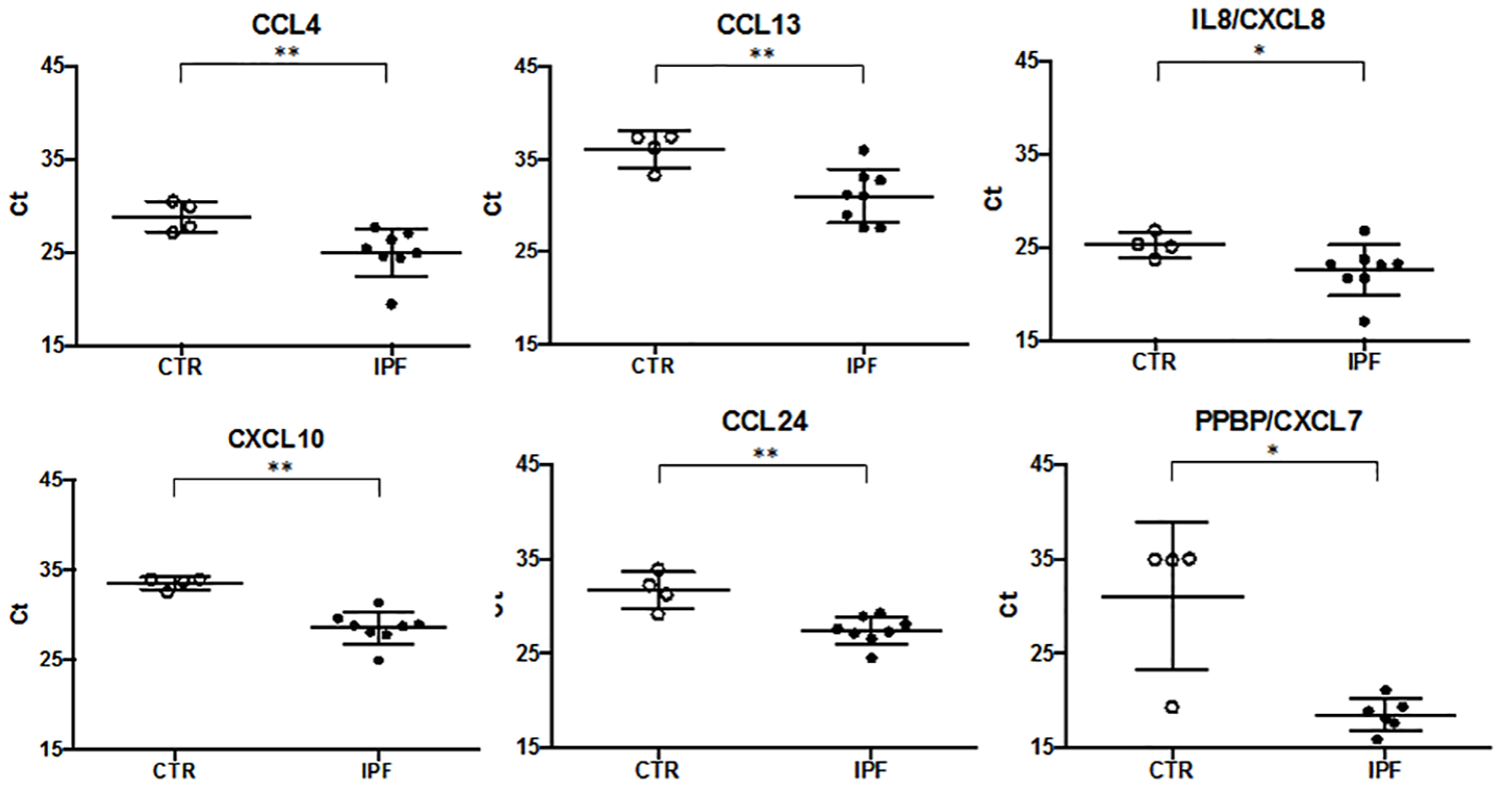
RNA-seq differential gene expression validation by qRT-PCR. Relative expression of selected chemokines in IPF (N = 7, closed circles) vs. Healthy control (N = 4, open circles) BAL cells represented by normalized ΔCt value. ΔCt value was normalized to the GNB2L1 housekeeping gene expression by subtracting GNB2L1 raw Ct from the selected gene raw Ct and by further adding the absolute GNB2L1 minimum value (the same for all samples) to the gene ΔCt. Lower cycle values correspond to higher expression level. Mann-Whitney test (*) and (**) denote statistical significance (P < 0.05, P < 0.01, respectively) vs. control.

**Fig 4:**
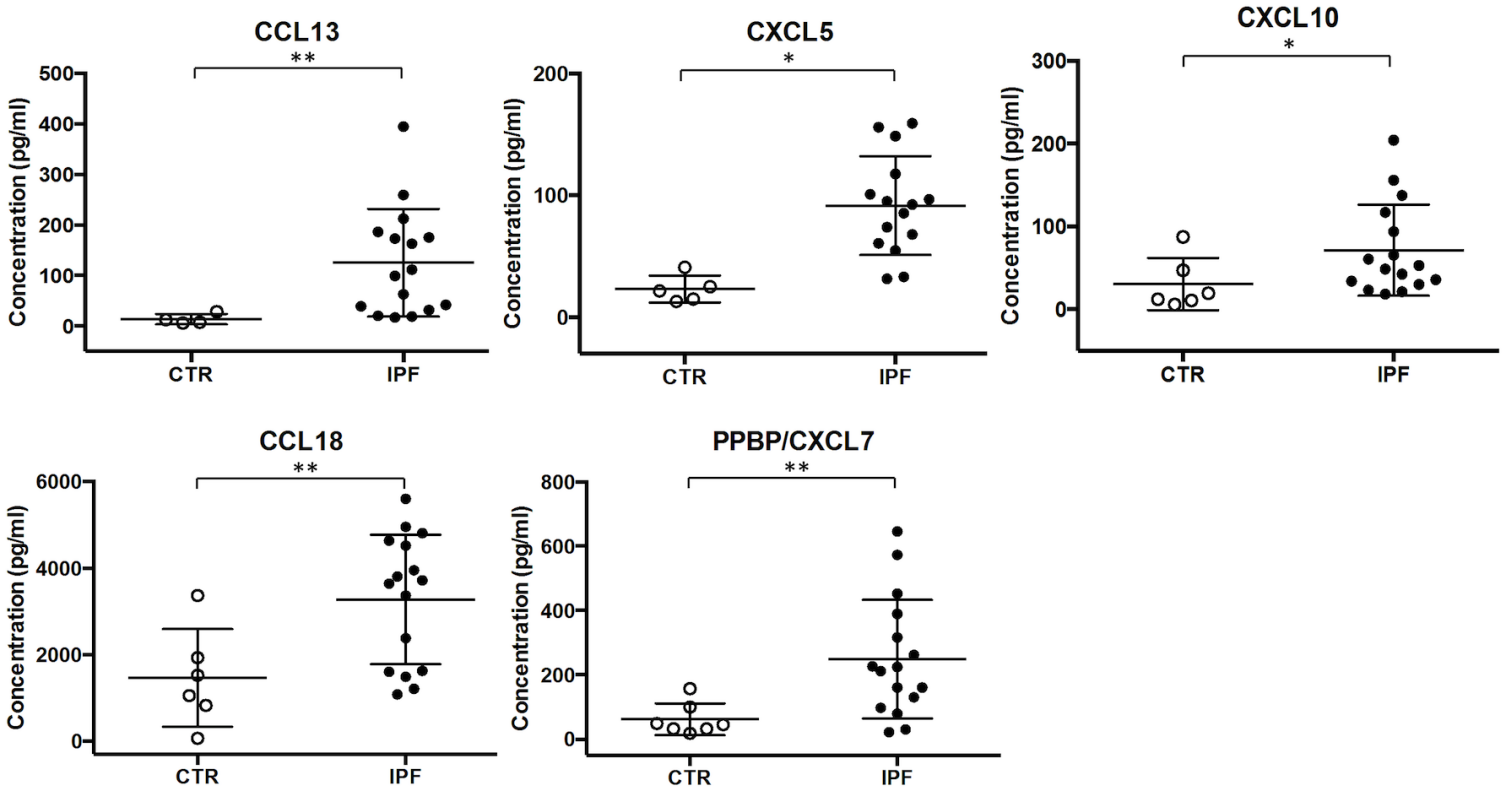
Quantification of BAL fluid chemokine levels by protein immunochemistry. Chemokines were measured in un-concentrated BAL fluid by protein immunochemistry on IPF affected individuals (closed circles) and Healthy Controls (open circles). CXCL-5 was quantified using the Chemokine Human 5-Plex Panel II for the Luminex 100/200 platform. PPBP/CXCL-7, CXCL-10, CCL13 and CCL18 were measured using enzyme-linked immunosorbent assay (ELISA) kits. Mann-Whitney test (*) and (**) denote statistical significance (P < 0.05, P < 0.01, respectively) vs. control.

Similarly, in the KEGG Pathway analysis the “Cytokine-cytokine receptor interaction”, “Chemokine signaling” and “TNF signaling” pathways were identified as most significantly overrepresented. These pathways comprised differentially expressed cytokine and chemokine genes largely overlapping with those identified by GO Biological Process and Molecular Function analysis (Table 2). They included CXC chemokines (CXCL2, CXCL3, CXCL5, CXCL7/PPBP, CXCL8/IL8, CXCL10), CC chemokines (CCL2, CCL4, CCL13, CCL18, CCL20, CCL24), the interleukin-1 receptor antagonist (IL1RN), and the NFKB inhibitor alpha (NFKBIA). Chemokine gene up-regulation was confirmed by quantitative PCR analysis of the CCL4, CXCL10, CCL13, CCL24, IL8/CXCL8, PPBP/CXCL7 genes using BAL cell lysates (Fig 3). Protein expression was assessed for the CXCL5, CXCL10, CCL13, CCL18, and PPBP/CXCL7 genes using BAL fluid immunochemistry (Fig 4).

In addition, over-representation of the GO Molecular Function “Haptoglobin binding” suggested ectopic expression of the hemoglobin genes HBA1, HBA2 and HBB by BAL cells. We thus compared the IPF alveolar macrophage differentially expressed genes (Table A in S1 File) with gene lists derived from published studies identifying macrophage activation patterns, including the “classic” pro-inflammatory M1, the “alternative”, anti-inflammatory/ pro-repair M2 and the chronic inflammatory M^TPP^–type activation patterns. Alveolar macrophage differentially expressed genes were concurrently, and significantly (p<0.05), enriched in M1 (chi-square = 4.8657, Pvalue = 0.0273), M2 (chi-square = 11.3596, Pvalue = 0.0007) as well as in M^TPP^-type activation genes (chi-square = 14.9939, Pvalue = 0.0001).

### *In vitro* modeling of iron-induced alveolar macrophage activation

**Fig 5:**
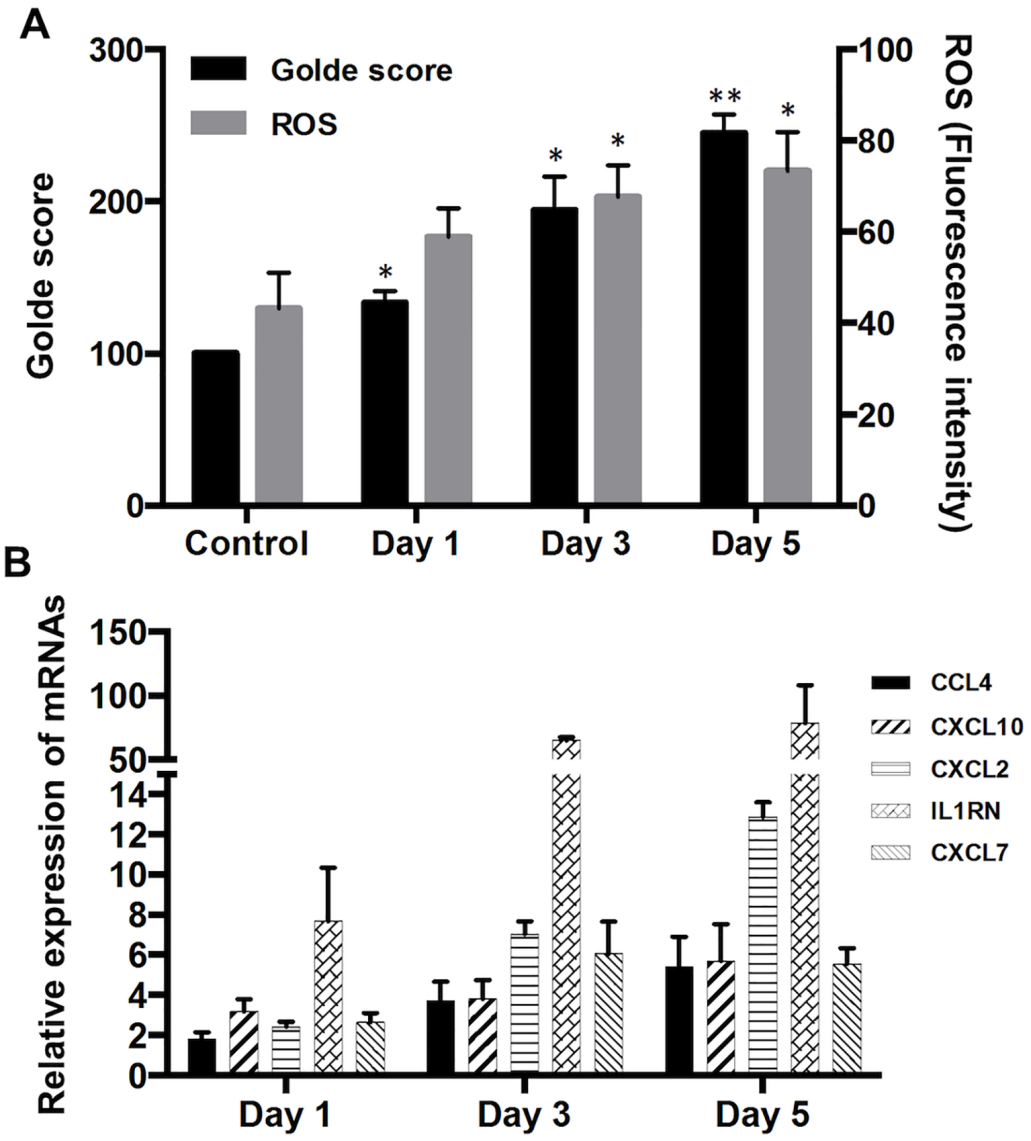
FAC-treated murine alveolar macrophage cells, MH-S. Murine alveolar macrophage cells (MH-S, ATCC CRL-2019) derived from a BALB/C strain mouse were incubated with 100 μM of Ferric ammonium citrate (FAC) for 1, 3, and 5 days. *A*. Shown are iron and ROS levels in FAC-treated cells. *B*. Shown are relative mRNA expression levels of selected chemokines/cytokines in FAC-treated mouse alveolar macrophages examined by qRT-PCR. Relative expression levels of the cytokines were normalized to the GNB2L1 housekeeping gene and calculated using the 2^ΔΔCt method. Mann-Whitney test (*) and (**) denote statistical significance (P < 0.05, P < 0.01, respectively) vs. control.

*In vitro* iron-exposed MH-S murine immortalized alveolar macrophages accumulated iron. They also generated increased ROS levels (Fig 5A), which were inhibited by iron chelation (iron alone = 70.6±4.7 CM-H2DCFDA fluorescence units; iron + DFP 100 μM = 38.6±6.5, P-value <0.05) (Table C in S1 File).

In addition, qPCR analysis of iron-laden MH-S macrophage gene expression showed that *in vitro* iron exposure was followed by a statistically significant dose (not shown) and time dependent increase in the expression of the selected M1, M2 and M^TPP^-type chemokine and cytokine genes (Fig 5B).

## Discussion

Consistent with the selection of subjects with BAL with >80% alveolar macrophages, differential cell counts showed 86.1 ± 3.84% alveolar macrophages in IPF patients and 83.4 ±7.68 in healthy controls (Table 1). Accordingly, the inspection of RNA sequence read counts (Table D in S1 File) of macrophage markers CD169, CD11b, CD14 and CD206 show high numbers of mRNA reads, while neutrophil gene markers MPO, LCN2, LTF and ELANE show very low reads numbers, in average about 50-fold lower that macrophage markers. Similarly low reads numbers were seen for the T-lymphocyte markers CD3- gamma, delta, epsilon and zeta genes (data not shown), thus suggesting that the data described above are representative of the alveolar macrophage cell population.

Unbiased gene expression analysis by RNA sequencing of IPF unstimulated BAL cells shows significant enrichment of Gene Ontology terms and KEGG pathways comprising overlapping sets of upregulated differentially expressed chemokine and cytokine genes that delineate an alveolar macrophage population expressing pro-inflammatory M1 and anti-inflammatory/pro-repair M2 activation phenotypes. This finding extends the observations of previous IPF BAL studies that reported increased levels of pro-inflammatory or pro-repair chemokine and cytokine expression/production by alveolar macrophages [3, 6]. We found that the “Cytokine-cytokine receptor interaction”, “Chemokine signaling” and “TNF signaling” pathways included chemokine genes typical of M1 and M2 macrophage activation. In addition, these pathways also included genes typical of M^TPP^ activated macrophages [30], i.e. neutrophil-attracting and angiogenic ELR+ CXC chemokine genes. These chemokines have been previously implicated in IPF exacerbation, vascular remodeling and fibrogenesis [32]. Finally, the above macrophage activation pattern observed *ex-vivo* in IPF patients was replicated in the murine alveolar macrophage MH-S cell line upon *in vitro* exposure to iron. Similar to IPF BAL cells evaluated *ex-vivo*, MH-S macrophages showed, together with iron stain positivity and iron-induced ROS generation, concomitant increased expression of selected M1, M2 and M^TPP^-type cytokine/chemokines genes. The data thus show that the IPF BAL cells express a composite activation pattern, remindful of the “unrestrained” macrophage activation pattern induced by iron accumulation and oxidative stress in disease models of tissue hemorrhage [14–16].

Iron accumulation in the lower respiratory tract can be observed consequent to aging although Prussian blue stained ferritin/hemosiderin appear to accumulate less than total iron [33] and both hemosiderin iron and total iron accumulation are associated with tobacco smoke [34]. Finally, hemosiderin iron accumulation is associated with alveolar hemorrhage. In this case it may be transient and non-harmful, as in autoimmune forms of idiopathic pulmonary hemosiderosis [35], or damaging as in UIP-like pulmonary fibrosis consequent to lung hemorrhage complicating coumarin anticoagulation [36]. Notably, iron accumulation has been observed in pulmonary veno-occlusive disease, where iron stain-positive alveolar macrophages are considered a sign of occult alveolar hemorrhage [37]. Similarly, in IPF patient, they are a sign of progression of pulmonary hypertension [9–13]. Finally, autopsy studies have described pulmonary hemorrhage and pulmonary thromboembolism, together with diffuse alveolar damage, as features of IPF exacerbation [38].

Pulmonary iron overload has been implicated in the pathogenesis of chronic obstructive pulmonary disease (COPD), a chronic disorder associated with tobacco smoke exposure whereby patients with advanced disease show alveolar macrophages with increased accumulation of iron [39]. Ferritin, whose expression in alveolar macrophages is capable of reducing iron-induced generation of oxygen radicals [40] has been shown by PCR analysis to be markedly upregulated in COPD [41]. The data in this study indicate that both ferritin-L and ferritin-H are expressed at the same ratio and level in IPF patients as in controls (data not shown). It is worth noticing that iron-dependent generation of oxygen radicals in IPF BAL macrophages has been associated with the carriage of the H63D allelic variant of the HFE hemochromatosis gene [17]. The H63D variant has been linked to increased risk of pulmonary fibrosis in cancer patients treated with bleomycin [42] and, interestingly, of increased risk of leg ulcers in varicose vein affected individuals [43], thus suggesting that dysregulated iron handling may play a role in macrophage activation in IPF. As diverse pathophysiological conditions, environmental factors and aging are implicated in iron accumulation in the lung, further studies are needed to gain better understanding of the regulation of iron transport and storage and its implications for macrophage pro-inflammatory, pro-repair and angiogenic response to excess iron in IPF lung.

## Supporting information

S1 File Supplementary tables. (DOCX)

S1 Fig Evaluation of subject’ age impact upon ferritin/hemosiderin accumulation in alveolar macrophages. (TIF)

S2 Fig Differential expression of hemoglobin genes in IPF BAL macrophages. (TIF)
