# Correction: Bronchoalveolar lavage (BAL) cells in idiopathic pulmonary fibrosis express a complex pro-inflammatory, pro-repair, angiogenic activation pattern, likely associated with macrophage iron accumulation

**DOI:** 10.1371/journal.pone.0197794

**Published:** 2018-05-17

**Authors:** The PLOS ONE Staff

There are errors in the Funding section. The correct funding information is as follows: This study was supported by University of Florida Departmental funds (UF project #00127916(C.S.) and #00045513(M.L.B)), and European Brain Research Institute funds (PAINCAGE FP7 #603191 (I.A.)). The funders had no role in study design, data collection and analysis, decision to publish, or preparation of the manuscript.

The publisher apologizes for the error.
