# Supplementary material for: Bronchoalveolar lavage (BAL) cells in idiopathic pulmonary fibrosis express a complex pro-inflammatory, pro-repair, angiogenic activation pattern, likely associated with macrophage iron accumulation
Source: PLoS One. 2018 Apr 12;13(4):e0194803. doi: 10.1371/journal.pone.0194803 (PMC5896901; doi:10.1371/journal.pone.0194803)
Supplement: S1 File — (DOCX) [file pone.0194803.s001.docx]

**Supporting Information**

**Evaluation of subject’ age impact upon ferritin/hemosiderin accumulation in alveolar macrophages**

The impact of age upon intracellular ferritin/hemosiderin accumulation in alveolar macrophages was evaluated using BAL cytopreparations obtained from an extended population of healthy control and IPF affected subjects. BAL cytopreps were stained with the Prussian blue stain and the Gold score was assessed as described [1] to assess the correlation between age and alveolar macrophage iron accumulation (S1 Fig). This extended population was comprised of 24 healthy individuals (seven of the control subjects were already included in the RNA sequence population) and 59 IPF affected individuals (16 of the IPF affected individuals were already included in the present study). Control subjects (11 non-smokers and 13 current smokers, 13 females and 11 males, average age=55.4±7.7), all with normal pulmonary function (FVC=112.4±12.8%, DLCO=90.17±11.74 %), were enrolled in this study, or in previous BAL studies after informed consent was obtained. For a subpopulation of 10 individuals the consent was approved by the regional ethics committee in Stockholm, Sweden, No 2006/959-31/1, as reported by Forsslund H et a. [2]. The remaining subjects were enrolled under an investigational protocol of the University of Tor Vergata, Rome (Policlinico Tor Vergata Ethics Committee, Rome, Italy: SCS/REDD/08 n.123/08).

BAL cell differential counts were: macrophages 87.0±7.7%, neutrophils 2.9±2.7%, lymphocytes 7.9±5.7%, eosinophils 0.7±1.4%, basophils 0%. IPF affected individuals (24 non- smokers and 35 current and former smokers, 18 females and 41 males, average age=67.9±8.9; FVC=74.6±25.6%, DLCO=57.0±63.2%) underwent diagnostic BAL after informed consent, as previously reported in this manuscript and as previously reported by Sangiuolo et al [3]. BAL cell differential counts were: macrophages 71.8±17.7% (P-value=0.0001, Unpaired T test, compared to Controls), neutrophils 15.6±14.8% (P-value=0.0001), lymphocytes 6.2±6% (P-value=0.23), eosinophils 3.8±6.3% (P-value=0,019), basophils 0.2±0.1% (P-value=0.31).

**Expression of Hemoglobin genes by BAL macrophages in IPF**

To validate BAL cell differential expression of hemoglobin genes obtained by RNA sequencing, BAL cell data were compared with public data of RNA sequencing of human red blood cell (Kabanova S. et al., Complete array dataset deposited in the Gene Expression Omnibus database (accession number – GSE3674) [4]). Normalized Reads Per Kilobase per Millions mapped (RPKM) reads were compared for the HBB, HBA1, HBA2 and HBD genes, using the relative differences between HBA1, HBA2, HBB and HBD (S2 Fig) [5, 6].

**RNA sequence CPM (Counts per Million) expression values**

Data are aggregated per gene symbol and were normalized by the RUVg method using the R Bioconductor package RUVSeq (S4 Table).

**Reference**

1. Puxeddu E, Comandini A, Cavalli F, Pezzuto G, D'Ambrosio C, Senis L, et al. Iron laden macrophages in idiopathic pulmonary fibrosis: the telltale of occult alveolar hemorrhage? Pulm Pharmacol Ther. 2014;28(1):35-40. doi: 10.1016/j.pupt.2013.12.002. PubMed PMID: 24365112.

2. Forsslund H, Mikko M, Karimi R, Grunewald J, Wheelock AM, Wahlstrom J, et al. Distribution of T-cell subsets in BAL fluid of patients with mild to moderate COPD depends on current smoking status and not airway obstruction. Chest. 2014;145(4):711-22. doi: 10.1378/chest.13-0873. PubMed PMID: 24264182.

3. Sangiuolo F, Puxeddu E, Pezzuto G, Cavalli F, Longo G, Comandini A, et al. HFE gene variants and iron-induced oxygen radical generation in idiopathic pulmonary fibrosis. Eur Respir J. 2015;45(2):483-90. doi: 10.1183/09031936.00104814. PubMed PMID: 25504993.

4. Kabanova S, Kleinbongard P, Volkmer J, Andree B, Kelm M, Jax TW. Gene expression analysis of human red blood cells. Int J Med Sci. 2009;6(4):156-9. PubMed PMID: 19421340; PubMed Central PMCID: PMCPMC2677714.

5. Bhaskaran M, Chen H, Chen Z, Liu L. Hemoglobin is expressed in alveolar epithelial type II cells. Biochem Biophys Res Commun. 2005;333(4):1348-52. doi: 10.1016/j.bbrc.2005.06.042. PubMed PMID: 15979582; PubMed Central PMCID: PMCPMC1314978.

6. Liu W, Baker SS, Baker RD, Nowak NJ, Zhu L. Upregulation of hemoglobin expression by oxidative stress in hepatocytes and its implication in nonalcoholic steatohepatitis. PLoS One. 2011;6(9):e24363. doi: 10.1371/journal.pone.0024363. PubMed PMID: 21931690; PubMed Central PMCID: PMCPMC3171444.

**Supplementary Table A. Differentially expressed genes in IPF patients compared to healthy controls**

| **Gene symbol** | **Log2FC** | **FDR** | **Mean CTR RPKM** | **Mean IPF RPKM** |
| --- | --- | --- | --- | --- |
| HBA1 | 3.491 | 3.84E-04 | 1.82 | 20.02 |
| HBA2 | 3.317 | 6.43E-04 | 1.94 | 18.94 |
| HBB | 3.144 | 1.10E-03 | 1.15 | 10.29 |
| PPBP | 2.755 | 8.24E-06 | 1.67 | 15.74 |
| CCL4 | 2.227 | 8.61E-03 | 2.24 | 9.92 |
| CXCL2 | 2.099 | 4.64E-04 | 3.75 | 14.78 |
| IL8 | 2.018 | 3.29E-02 | 7.14 | 20.79 |
| GPC2 | 1.985 | 3.31E-02 | 5.48 | 27.87 |
| SPP1 | 1.978 | 4.18E-03 | 10.63 | 47.57 |
| CCL20 | 1.886 | 4.17E-02 | 2.69 | 9.93 |
| G0S2 | 1.819 | 9.13E-04 | 1.90 | 6.74 |
| CCL24 | 1.772 | 8.53E-04 | 3.03 | 10.61 |
| LYSMD1 | 1.671 | 3.87E-02 | 22.33 | 51.03 |
| CXCL5 | 1.627 | 1.12E-03 | 3.77 | 13.89 |
| IFI27 | 1.506 | 2.92E-02 | 4.70 | 13.40 |
| NDUFAB1 | 1.501 | 3.88E-02 | 34.98 | 67.67 |
| IER3 | 1.467 | 3.77E-02 | 1.67 | 4.47 |
| FAM228A | 1.464 | 4.36E-02 | 15.99 | 28.95 |
| CXCL3 | 1.451 | 2.41E-02 | 4.46 | 11.05 |
| PLK1S1 | 1.428 | 4.74E-02 | 72.89 | 130.40 |
| S100P | 1.427 | 1.00E-06 | 1.83 | 4.85 |
| DNAJC1 | 1.406 | 2.49E-02 | 10.29 | 19.40 |
| RPF2 | 1.406 | 2.57E-02 | 42.53 | 78.09 |
| CCL18 | 1.403 | 3.94E-03 | 116.64 | 213.68 |
| C3orf38 | 1.396 | 4.14E-02 | 26.74 | 45.56 |
| HOXC9 | 1.396 | 3.01E-02 | 4.81 | 9.98 |
| AADAC | 1.377 | 4.45E-02 | 9.63 | 19.40 |
| DUSP2 | 1.353 | 3.02E-02 | 0.84 | 2.13 |
| IRF2BP1 | 1.337 | 3.83E-02 | 7.76 | 14.95 |
| IL20RB | 1.320 | 3.96E-02 | 20.17 | 35.61 |
| SLC38A3 | 1.317 | 2.41E-02 | 6.60 | 12.99 |
| CCL13 | 1.316 | 1.68E-03 | 1.28 | 3.20 |
| FAM124B | 1.295 | 4.53E-02 | 22.95 | 39.70 |
| FAM71E1 | 1.280 | 4.39E-02 | 12.39 | 23.75 |
| AVIL | 1.268 | 4.26E-02 | 12.51 | 24.46 |
| OAF | 1.267 | 2.31E-02 | 2.58 | 5.38 |
| RBPJL | 1.266 | 3.79E-02 | 9.09 | 18.14 |
| C1orf87 | 1.266 | 4.62E-02 | 22.24 | 37.44 |
| CTSE | 1.254 | 4.78E-02 | 31.40 | 51.97 |
| CD8A | 1.250 | 2.49E-02 | 11.37 | 23.88 |
| PIAS4 | 1.247 | 3.81E-02 | 11.43 | 19.04 |
| CCL2 | 1.242 | 4.97E-02 | 5.32 | 9.67 |
| STRA13 | 1.233 | 2.08E-02 | 33.43 | 59.61 |
| SUOX | 1.230 | 4.49E-02 | 63.17 | 102.05 |
| ABRACL | 1.228 | 3.87E-02 | 59.86 | 94.68 |
| MRGPRX2 | 1.210 | 4.82E-02 | 15.28 | 25.80 |
| LNX1 | 1.207 | 3.98E-02 | 18.95 | 31.56 |
| LSMEM2 | 1.200 | 2.08E-02 | 3.35 | 7.22 |
| TPCN1 | 1.199 | 3.35E-02 | 13.35 | 23.60 |
| NNMT | 1.192 | 3.21E-02 | 1.40 | 3.16 |
| CHST8 | 1.190 | 4.28E-02 | 13.87 | 26.44 |
| TMEM59L | 1.189 | 3.37E-02 | 8.69 | 16.21 |
| ZCWPW2 | 1.189 | 4.77E-02 | 24.82 | 40.05 |
| UPB1 | 1.156 | 3.35E-02 | 10.14 | 17.23 |
| TBX5 | 1.149 | 4.00E-02 | 24.31 | 40.29 |
| MARVELD2 | 1.137 | 2.93E-02 | 15.31 | 30.56 |
| FAM170A | 1.129 | 3.81E-02 | 26.10 | 42.84 |
| NFKBIA | 1.128 | 4.98E-02 | 8.61 | 18.32 |
| WDR70 | 1.126 | 3.27E-02 | 7.75 | 14.10 |
| FABP4 | 1.108 | 4.11E-03 | 67.32 | 98.65 |
| PI3 | 1.098 | 3.30E-03 | 0.88 | 1.89 |
| IQCF5 | 1.083 | 3.18E-03 | 0.54 | 1.18 |
| HIST1H3G | 1.077 | 1.33E-04 | 1.15 | 2.37 |
| MED19 | 1.076 | 4.34E-02 | 21.03 | 33.29 |
| CLC | 1.063 | 4.57E-03 | 0.97 | 1.98 |
| DEFB1 | 1.050 | 2.62E-02 | 1.90 | 4.03 |
| IGFBP5 | 1.039 | 3.35E-02 | 6.09 | 9.88 |
| HIST1H3C | 1.034 | 1.52E-06 | 3.00 | 5.87 |
| VCL | 1.027 | 3.77E-02 | 23.18 | 36.32 |
| SCGB3A1 | 1.022 | 4.99E-02 | 0.91 | 1.81 |
| HIST1H4B | 1.021 | 1.78E-03 | 1.11 | 2.10 |
| CXCL10 | 1.020 | 2.97E-03 | 1.19 | 2.40 |
| TFEB | 1.010 | 3.81E-02 | 51.22 | 81.90 |
| HIST1H2AJ | 1.009 | 1.94E-04 | 3.37 | 6.78 |
| IL1RN | 1.008 | 1.23E-04 | 28.38 | 56.28 |
| LPL | 1.008 | 1.41E-03 | 11.31 | 26.11 |
| GJA4 | 1.003 | 3.52E-02 | 11.13 | 19.51 |
| PCYT1A | 1.002 | 3.52E-02 | 28.64 | 41.52 |
| ATP6V0D2 | -1.009 | 2.21E-04 | 6.94 | 3.39 |
| SEMA6B | -1.145 | 2.45E-03 | 3.93 | 1.53 |
| DCSTAMP | -1.225 | 1.00E-06 | 14.81 | 5.59 |
| DNASE2B | -1.226 | 1.00E-06 | 19.40 | 7.24 |
| EVI2B | -1.228 | 1.69E-05 | 145.70 | 71.09 |
| UCHL1 | -1.256 | 1.19E-02 | 3.83 | 1.51 |
| NUPR1 | -1.465 | 1.00E-06 | 74.17 | 27.06 |
| CES1 | -1.468 | 1.69E-05 | 113.35 | 34.70 |
| MTRNR2L8 | -1.834 | 1.34E-04 | 32.72 | 9.84 |
| CYP1B1 | -1.969 | 3.42E-04 | 6.25 | 1.40 |

**Supplementary Table B. Primer information**

| **Gene ID** | **Forward primer (5' to 3')** | **Reverse primer (5' to 3')** |
| --- | --- | --- |
| CCL4 | CCCAGCCAGCTGTGGTATT | GCAGCTCAGTTCAGTTCCAG |
| CXCL10 | CAGCAGAGGAACCTCCAGTC | CAGCGTACAGTTCTAGAGAGAGG |
| CCL13 | ATGTGATCACCACCAGCAGG | ATTCTGGACCCACTTCTCCT |
| CCL24 | GTGTCTGTGCCCACCACAT | GACCACTCGGTTCTCAGGAA |
| PPBP/CXCL7 | GCTGCTGACTGCTCTGGC | TCCCATCCTTCAGTGTGGC |
| IL-8/CXCL8 | AAGAACTGAGAGTGATTGAGAGTGG | GTTTTCCTTGGGGTCCAGACA |
| GNB2L1 | GAGTGTGGCCTTCTCCTCTG | GCTTGCAGTTAGCCAGGTTC |
| mGnb2l1 | GTCTGCAAGTACACGGTCCA | ATCCGCAGGAGACGATGATA |
| mCxcl2 | AGTGAACTGCGCTGTCAATG | TCCAGGTCAGTTAGCCTTGC |
| mIl1rn | GTGCCAAGTCTGGAGATGAT | GGACAGGCAGCTGACTCAA |
| mPpbp/Cxcl7 | TGGAAGTGATAGCCACACTGA | GAAGCAGCTGGTCAGTAACCTT |
| mCxcl10 | ATCAGCACCATGAACCCAAG | GTGGCAATGATCTCAACACG |
| mCcl4 | TTCTGTGCTCCAGGGTTCTC | GACTGCTGGTCTCATAGTAATCCA |

**Supplementary Table C. ROS in FAC and deferiprone-treated murine alveolar macrophage cells, MH-S**

| **Samples** | **ROS** |
| --- | --- |
| Control | 54.8±3.8 |
| FAC (30 μM)-treated cells | 70.6±4.7 |
| FAC (30 μM) and Deferiprone (100 μM )-treated cells | 49.3±1.7 |
| FAC (30 μM) and Deferiprone (150 μM )-treated cells | 38.6±6.5 |
