# Supplementary figures and images for: Bronchoalveolar lavage (BAL) cells in idiopathic pulmonary fibrosis express a complex pro-inflammatory, pro-repair, angiogenic activation pattern, likely associated with macrophage iron accumulation
Source: PLoS One. 2018 Apr 12;13(4):e0194803. doi: 10.1371/journal.pone.0194803 (PMC5896901; doi:10.1371/journal.pone.0194803)

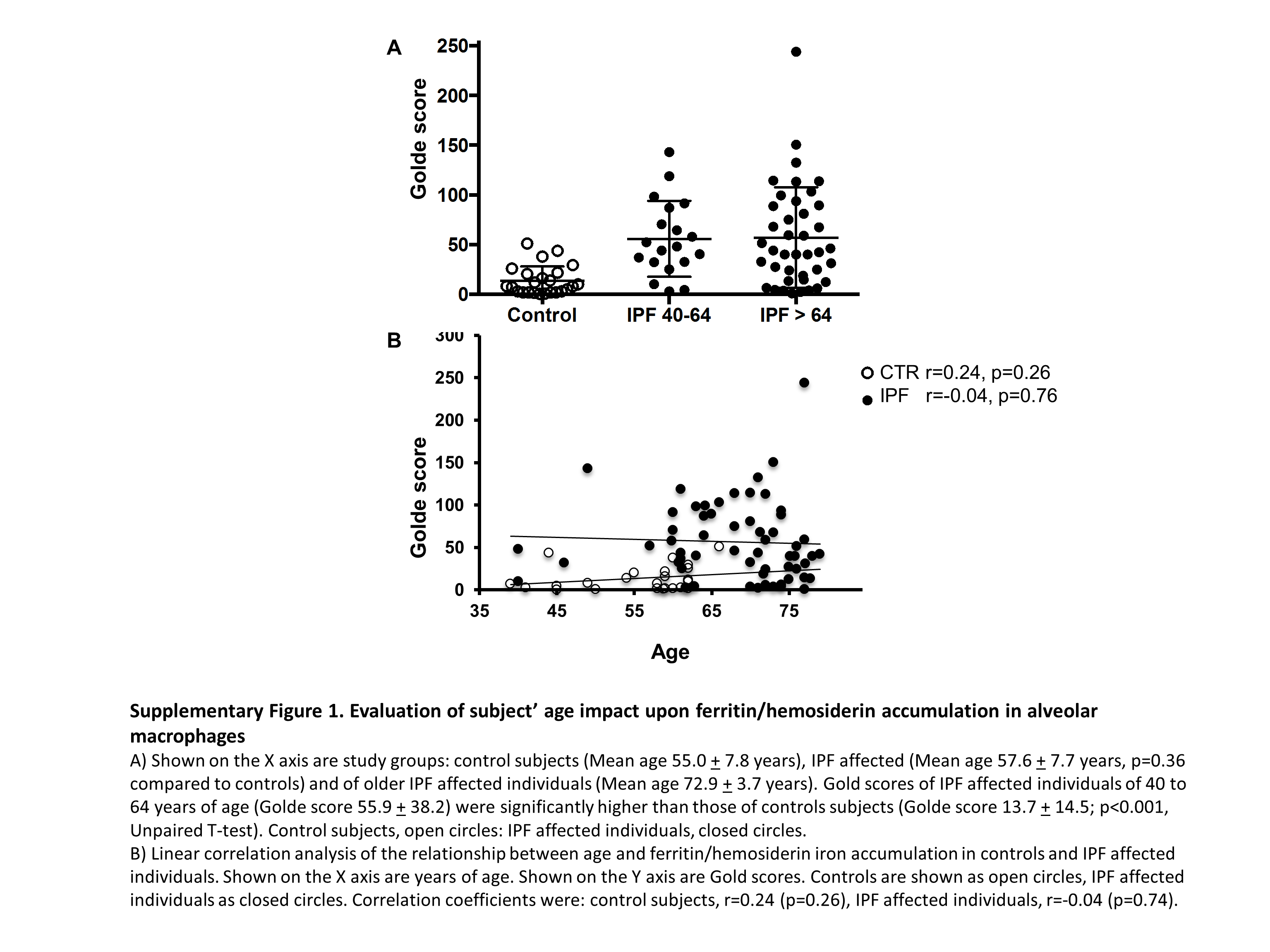

Supplement: S1 Fig — (TIF) [file pone.0194803.s002.TIF]

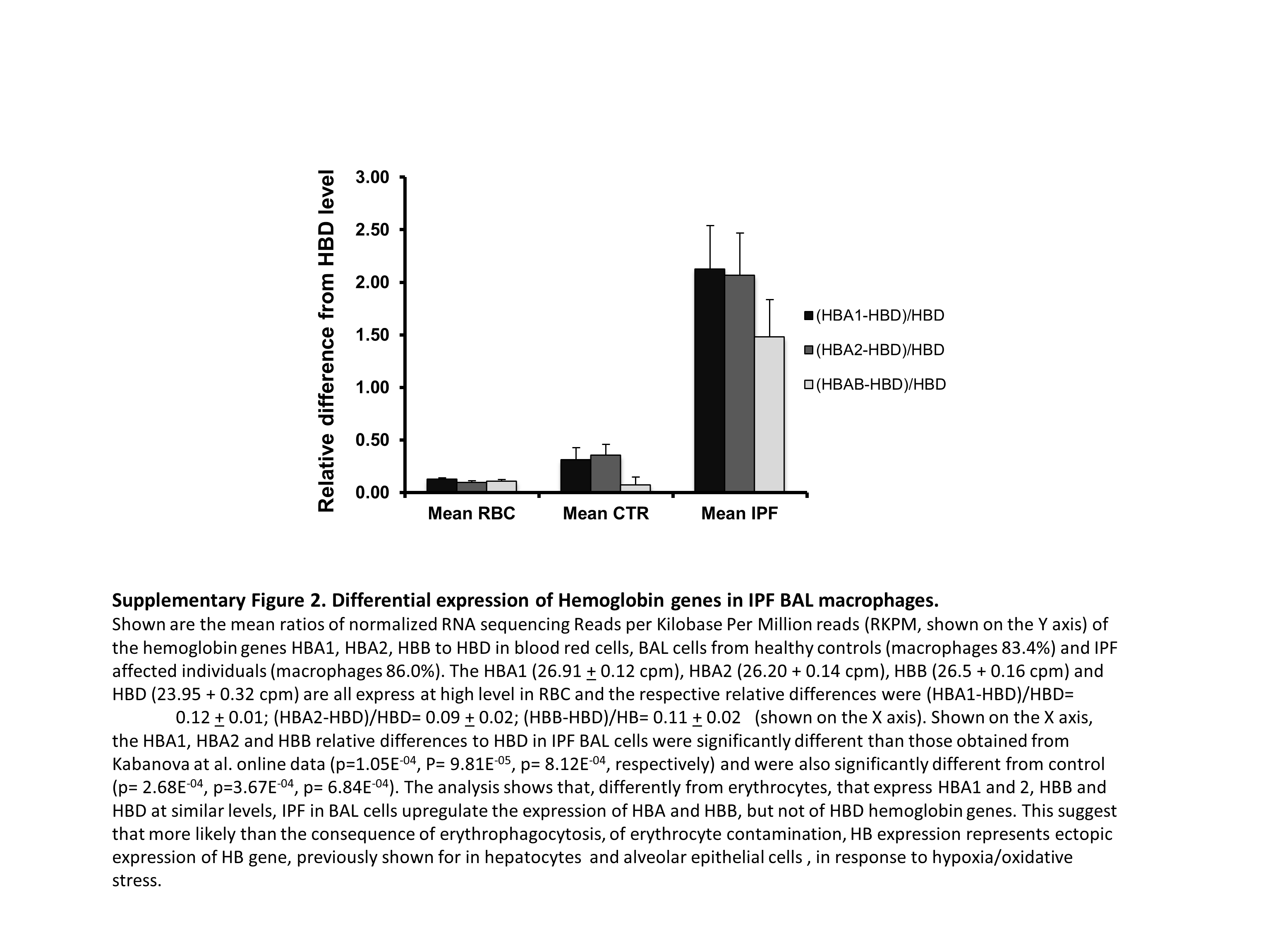

Supplement: S2 Fig — (TIF) [file pone.0194803.s003.TIF]
